# Improved Jacobian matrix estimation applied to snake robots

**DOI:** 10.3389/frobt.2023.1190349

**Published:** 2023-05-25

**Authors:** Jostein Løwer, Damiano Varagnolo, Øyvind Stavdahl

**Affiliations:** Department of Engineering Cybernetics, Norwegian University of Science and Technology, Trondheim, Norway

**Keywords:** snake robots, Kalman filter, Jacobian estimation, hyper-redundant robots, soft robots

## Abstract

Two manipulator Jacobian matrix estimators for constrained planar snake robots are developed and tested, which enables the implementation of Jacobian-based obstacle-aided locomotion (OAL) control schemes. These schemes use obstacles in the robot’s vicinity to obtain propulsion. The devised estimators infer manipulator Jacobians for constrained planar snake robots in situations where the positions and number of surrounding obstacle constraints might change or are not precisely known. The first proposed estimator is an adaptation of contemporary research in soft robots and builds on convex optimization. The second estimator builds on the unscented Kalman filter. By simulations, we evaluate and compare the two devised algorithms in terms of their statistical performance, execution times, and robustness to measurement noise. We find that both algorithms lead to Jacobian matrix estimates that are similarly useful to predict end-effector movements. However, the unscented filter approach requires significantly lower computing resources and is not poised by convergence issues displayed by the convex optimization-based method. We foresee that the estimators may have use in other fields of research, such as soft robotics and visual servoing. The estimators may also be adapted for use in general non-planar snake robots.

## 1 Introduction

Snake robots are mechanisms designed to mimic biological snakes, which aspire to inherit the robustness and stability of biological snake locomotion. Like their biological counterparts, and as explained in detail in Ariizumi and Matsuno (2017); Gray (1946), mechanical snakes move using an array of different propulsion techniques, such as lateral undulation, sinus lifting, and sidewinding. In principle, this makes snake robots suitable for moving and adapting to unknown and challenging environments, such as in rubble following landslides or collapsed buildings. As of now, this is largely an unrealized potential. Many experimental systems for obstacle-aided locomotion (OAL) adapt to the environment in an implicit or heuristical manner only, with little utilization of mechanical sensor information. In contrast, the present work is part of an effort to achieve efficient, robust, and intelligent locomotor behavior by exploiting information about the geometry and mechanical properties of the surroundings of the robot.

To do so, a generic strategy consists of calculating and then exploiting the *manipulator Jacobian* (or simply *Jacobian*) of the system. The Jacobian is a matrix which relates the robot joint velocities to its end-effector velocities through a linear transformation parameterized by the joint states (see Section 3 for more details). In many robot systems, the kinematics of the robot is known and time-invariant, which makes it possible to compute the Jacobian analytically. In the case of snake robots, however, computing the Jacobian is a much more involved task, partly because it depends on the continually changing configuration of contact points between the robot and the environment. In snake robots, the Jacobian matrix gives a relation between the joint speeds and the velocity of the head. This information may prove useful when designing locomotion strategies for snake robots, in the same way that Jacobian is essential in motion planning for robotic manipulators. This provokes the need for research on how to effectively estimate Jacobian matrices in constrained snake robots, ideally in real-time.

The present paper addresses this problem, but specifically in the case of planar snake robots, i.e., ones that are intended to navigate on a smooth, two-dimensional surface, with obstacles that constrain the robot’s movements. Planar snake robots are configured such that the axes of rotation of all joints are perpendicular to the ground plane. Therefore, they are unable to lift parts of their body off this plane, and thus cannot utilize gaits such as sidewinding and sinus lifting. Because of this, planar snake robots rely on either anisotropic friction between their body and the ground plane or its macroscopic equivalent: contact with obstacles, for propulsion. Planar snake robots have limited practical use, but their motion perfectly resembles that of a general 3D snake robot exhibiting pure lateral undulation (i.e., with no lifting action) on a flat surface containing obstacles. Thus, they lend themselves to studying this particular mode of propulsion. Furthermore, results based on planar motion may create a foundation on which research generalized to non-planar scenarios can be performed, and the chosen platform thus enables basal research into OAL and related subjects.

Planar snake robots share similarities with robot manipulators, in the sense that they both are constituted of primarily rotational joints and rigid links. However, they differ in the following aspects:

1. A planar snake robot is continually in contact with the surface underneath the robot. This introduces friction between the robot and the surface.
2. Most robot manipulators are grounded, in the sense that a base coordinate frame is typically fixed in the world frame. In contrast, generally no part of a planar snake robot is fixed in relation to the world frame.
3. A typical manipulator is intended to interact with its environment only through its end-effector; thus, its kinematic equations have a constant structure. In contrast, a snake robot is intended to interact with its environment at any point of any link. The corresponding constraints cause structural changes in the kinematics of the robot/environment system as the robot comes into contact with new obstacles or departs from obstacles it was previously in contact with.

**FIGURE 1:**
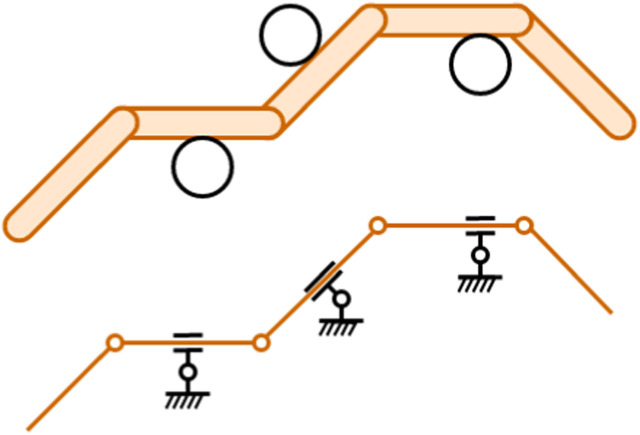
Top view of a 5-link planar snake robot (orange) in contact with three obstacles (black outline). The kinematic representation of the snake robot interacting with the obstacles is shown in the lower figure. Note how the obstacles can be modeled as a translational joint and a rotational joint attached to the world frame.

The last aspect is especially important as we discuss the motivation for this paper. Figure 1 shows how a snake robot in contact with its environment might be modeled as a kinematic chain, using pairs of translational and rotational joints fixed in the world frame to model the obstacles. The kinematics of such a model is examined in detail in Gravdahl et al. (2022); Liljeback et al. (2010) and will not be treated further, as the Jacobian matrix estimation strategies proposed in this paper are completely independent of the model. As the snake moves through its environment, the number of obstacle constraints and their positions relative to the robot will change. With incomplete prior knowledge of the position, shape, and orientation of each obstacle, it is challenging to ascertain the constrained kinematics of the snake robot. Finding the Jacobian for a constrained planar snake robot is desirable from a control perspective, but due to the uncertain nature of the snake robot’s kinematics, finding the Jacobian in a closed form is challenging. This paper seeks to find an estimate $\hat{J}$ for the robot Jacobian, without the need for an exact model of the constrained kinematics of the system.

Estimating Jacobians for robot manipulators is an established and well-researched field. Different methods for Jacobian estimation are diligently used in the field of visual servoing (Wang et al., 2019; Hosoda and Asada, 1994; Kosmopoulos, 2011; Shademan et al., 2010), which serve as inspiration for the contributions in this paper. Similar methods have been applied for calibration of robotic stereo vision (Qian and Su, 2002). While this paper specifically addresses Jacobian estimation in planar snake robots, the endeavor of estimating Jacobians is highly relevant to other fields within robotics and is discussed further in Section 8.

## 2 Notation

**FIGURE 2:**
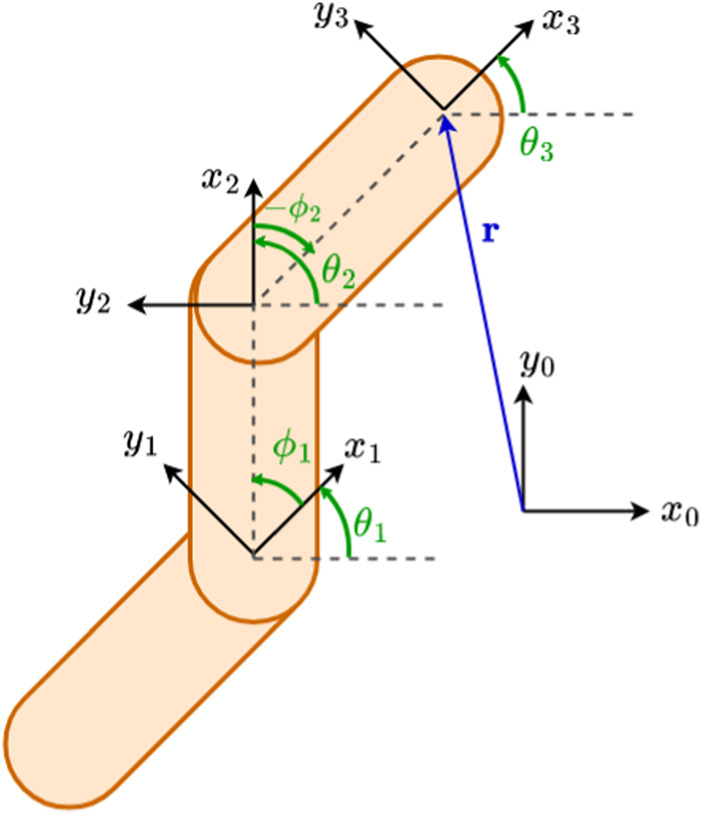
Schematic diagram for the computation of the kinematics of a simple 3-link planar snake robot as previously mentioned. The link angles *θ*_*i*_ and joint angles *ϕ*_*i*_ relate to the orientation of the local frames of the links.

The notation used for the remainder of the paper is inspired from Liljebäck et al. (2012). The kinematics characterizing a planar snake robot may be derived by inspecting Figure 2: a generic planar snake robot consisting of *N* links is composed of *N*_*j*_ = *N* − 1 joints, whose axes are oriented in the same direction. The robot is assumed to be embedded in a frame of reference denoted by (*x*_0_, *y*_0_). Each link of the robot has its own link local coordinate frame (*x*_*i*_, *y*_*i*_), where *i* is the link number. The local frames are oriented such that the *x*-axis forms a line between the axis of joints *i* and *i* − 1, and the *y*-axis points in the left transversal direction. The tail link of the robot is indexed as link 1 and the head as link *N*. As shown in the figure, the angle between the global axis *x*_0_ and the local axis *x*_*i*_, for *i* ∈ [1…*N*], is then denoted as *θ*_*i*_ and called the link angle of link *i*. The relative angles between adjacent links, i.e., the joint angles, are instead denoted as *ϕ*_*i*_ for *i* ∈ [1…*N*_*J*_]. It follows that the relation between the link angles and the joint angles is given by

$$\phi_{i}=\theta_{i+1}-\theta_{i}.$$ (1)

The vector containing the joint angles ***ϕ*** and vector containing the joint speeds $\dot{\phi }$ then are defined as

$$\begin{array}{cc} \phi & ={\left[\phi_{1},\phi_{2},\ldots ,\phi_{N_{j}}\right]}^{⊤}\\ \dot{\phi } & =\frac{d}{dt}\phi ={\left[{\dot{\phi }}_{1},{\dot{\phi }}_{2},\ldots ,{\dot{\phi }}_{N_{j}}\right]}^{⊤} \end{array}.$$

For the remainder of this paper, the superscript (⋅)^*n*^ denotes the iteration step of the algorithm being studied.

## 3 The manipulator Jacobian

Consider a generic robot manipulator with *N* rigid links and *N* − 1 joints, as shown in Figure 2. We denote the end-effector position as ***x***, the end-effector velocity as $\dot{x}$ , the joint angles as ***q***, and the joint velocities as $\dot{q}$ . The end-effector position is related to the joint angles by a forward kinematic function:

$$x=f\left(q\right)\;.$$ (2)

If a robot involves one or more rotational joints, the kinematic function ***f*** is often highly non-linear. For the purpose of developing automatic manipulation control schemes, it is of interest to determine how a set of joint velocities $\dot{q}$ will affect the velocity of the end-effector, $\dot{x}$ . This can be obtained by differentiating (2) with respect to time so that

$$\dot{x}=J\left(q\right)\dot{q}.$$ (3)

From an intuitive perspective, the Jacobian matrix ***J*** (***q***) corresponds to a parameterized linear transformation of the joint velocities $\dot{q}$ to the end-effector velocities $\dot{x}$ . The Jacobian is also essential in mapping between joint torques and tool point forces and torques. In the case that ***J*** (***q***) is invertible, this quantity can be used to compute a set of joint velocities $\dot{q}$ for any desired end-effector velocity $\dot{x}$ . In this case,

$$\dot{q}=J^{-1}\left(q\right)\dot{x}.$$ (4)

If ***J*** (***q***) is not invertible, then computing $\dot{q}$ as a function of $\dot{x}$ may be performed using other methods relying on the existence of ***J*** (***q***), such as constrained optimization, or by determining the Moore–Penrose inverse ***J*** ^+^ ***q***) for ***J*** (***q***).

In summary, the availability of ***J*** (***q***) is beneficial from a control perspective. For most robotics applications, the manipulator Jacobian ***J*** (***q***) can be found analytically as the kinematics of the robot is known. In the case of snake robots, the Jacobian relates the joint velocities to any of the snake robot’s state variables. We might not only be interested in the movement of the end-effector (the head link in the case of snake robots) but also in the motion of the remaining links in the robot since this information might be useful for activities such as low-level control or path planning. Furthermore, the correspondence between joint torques and contact forces has significant relevance for effective OAL, e.g., for minimizing obstacle-related friction, and avoidance or resolution of jam situations (*cf.* Liljeback et al., 2009).

As mentioned in the introduction and hinted in Figure 1, the Jacobian of a constrained snake robot depends on how and where the robot touches obstacles. In OAL situations, the configuration of such obstacles relative to the robot is continually changing, implying that the corresponding Jacobian is also time-varying. This introduces the problem of having to estimate it from field data as the locomotion is unfolding.

## 4 An optimization-based Jacobian matrix estimation approach

**FIGURE 3:**
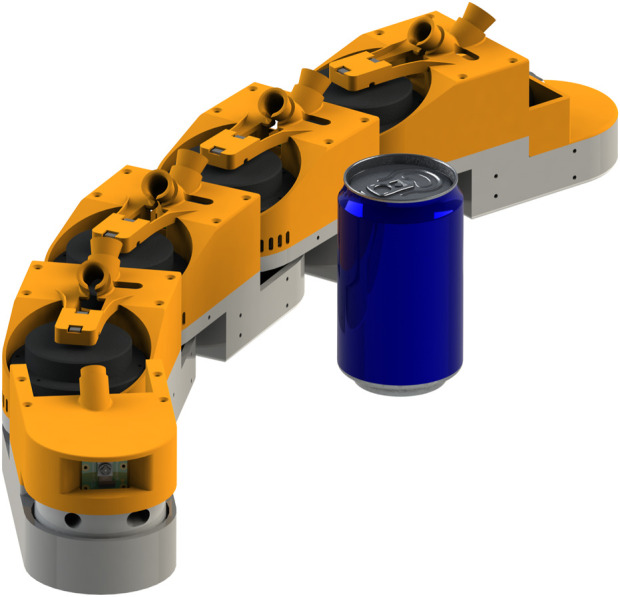
Simplified schematic of the control flow in the model-less control framework presented by Yip and Camarillo (2014). At each time step *n*, the scheme computes a current estimate ${\hat{J}}^{n}$ of the Jacobian, combines it with the desired trajectory ${\dot{x}}_{d}^{n}$ , determines the desired joints velocities ${\dot{q}}^{n}$ , and actuates them. The system then measures the trajectory of the robot ${\dot{x}}^{n}$ and uses it together with ${\dot{q}}^{n}$ to compute ${\hat{J}}^{n+1}$ , the estimated Jacobian, for the next time step.

The first estimator proposed in this paper is inspired from Yip and Camarillo (2014, 2016), where the authors present a framework for model-less control in soft robots. Soft robots share a property with planar snake robots, in that the exact kinematics of the robot is difficult to ascertain. The control framework considered in Yip and Camarillo (2014, 2016), schematized in Figure 3, relies on recursively estimating the Jacobian based on measurements of the soft robot’s control inputs and of the resulting end-effector movements. We adapt the model-less control framework to be usable for planar snake robots. The notation used in this section is consciously adapted to conform with the choices mentioned in Section 2. The joint states of the snake robot are given by the joint angles ***ϕ*** ≜ ***q***, and the end-effector position in the reference frame is given by the position of the head of the snake robot, given in world frame ***x*** ≜ ***r***. This paper explicitly considers using the position of the head of the robot ***r*** as the system output, a choice that most closely relates to the system output used in Yip and Camarillo (2014). In practice, one may choose different system outputs, even if this may add computational complexity. This could include the linear velocity of any of the remaining links or the angular velocities of the links. Given a current estimate of the Jacobian ${\hat{J}}^{n}$ , the current joint speeds ${\dot{\phi }}^{n}$ , and the current head velocity ***r*** ^*n*^, the modified Jacobian estimator for a snake robot is given by the optimization problem

$$\begin{array}{cc} \underset{{\hat{J}}^{n+1}}{\min}\; & {\left\|\Delta J\right\|}_{2}\\ s.t.\; & {\hat{J}}^{n+1}={\hat{J}}^{n}+\Delta J\\ & {\dot{r}}^{n}={\hat{J}}^{n+1}{\dot{\phi }}^{n}. \end{array}$$ (5)

Problem (5) can be interpreted as follows: find a minimal change Δ ***J*** in the current Jacobian ***J*** ^*t*^ so that the new Jacobian ***J*** ^*t*+1^ = ***J*** ^*t*^ + Δ ***J*** explains the relation between the measured values ${\dot{r}}^{t}$ and ${\dot{\phi }}^{t}$ . The Euclidean norm ${\left\|\cdot \right\|}_{2}$ is used as the metric for determining the magnitude of the change in the Jacobian. Because of this, (5) falls in the category of equality-constrained convex optimization (CO) problems for which many efficient numerical solvers exist (Kronqvist et al., 2019). Some care should be taken when using this method as (5) may have some undesirable properties under specific conditions. By definition, (5) is not guaranteed to have a unique (and global) optimum and can, under some conditions, have infinitely many or no solutions. An inherent issue with the Jacobian proposed in Yip and Camarillo (2014) is that if the actuators of the robot are stationary, solving the optimization problem does not lead to meaningful estimates. On inspection of the last constraint in (5), the estimated Jacobian ${\hat{J}}^{n+1}$ will uncontrollably diverge for sufficiently small values of $\dot{\phi }$ . In a real-life scenario, the measured values ${\dot{r}}^{t}$ and ${\dot{\phi }}^{t}$ will be noisy to some degree. An optimization-based formulation of the estimator makes it difficult to analytically find the statistical properties of the estimates (the statistical properties of the estimator $\hat{J}$ are not discussed in Yip and Camarillo (2014). A lack of statistical performance indices could lead to issues down-the-line if the estimates are used in, e.g., control strategies, as control strategies typically require information about the statistical properties of the estimate to infer qualities such as stability and convergence.

## 5 An unscented Kalman filter-based Jacobian matrix estimator

The unscented Kalman filter (UKF) approach is designed to perform state and parameter estimation on non-linear state-space problems by improving on the extended Kalman filter (EKF). The main difference between these two filters is the mechanism for propagating the error covariances: while the EKF relies on the linearization of the state-space model, the UKF relies on unscented transforms (Julier and Uhlmann, 2004; Wan and Van Der Merwe, 2000). The UKF has been shown to statistically outperform the EKF for a large subset of problems while exhibiting a similar or even reduced computational load. While both methods rely on an assumption that the underlying is locally linearizable around its state, the UKF outperforms the EKF in scenarios where the model nevertheless contains discrete non-linearities, owing to its use of the unscented transform. This is an essential property when performing estimation on a moving snake robot as it might come in contact with new obstacles or depart from obstacles it previously was in contact with. This will cause a discrete change in the system kinematics and, thus, also in its Jacobian.

Moreover, the UKF can be used as a model parameter estimation algorithm, the application of which is the primary interest in this paper. The general parameter estimation problem is stated by Wan and Van Der Merwe (2000) as

$$y^{n}=G\left(x^{n},w\right)\;,$$ (6)

where the non-linear map G(⋅) relates a system input ***x*** ^*n*^ to a system output ***y*** ^*n*^ parameterized by a vector ***w*** (note that Wan and Van Der Merwe (2000) uses a notation for which the state variable ***x*** has a different meaning than in our Sections 2 and 3).

Estimating the Jacobian ${\hat{J}}^{n+1}$ in (5) can then be formulated as a parameter estimation problem in which the input is $x^{n}={\dot{\phi }}^{n}$ , the output is $y^{n}={\dot{r}}^{n}$ , the parameter is $w={\hat{J}}^{n+1}$ , and the non-linear mapping G(⋅) is the matrix multiplication operation. This enables using a UKF-based approach to estimate ${\hat{J}}^{n+1}$ via reformulating the parameter estimation problem into the non-linear state-space representation

$$\begin{array}{cc} {\hat{J}}^{n+1} & ={\hat{J}}^{n}+\eta^{n}\\ {\dot{r}}^{n} & ={\hat{J}}^{n}{\dot{\phi }}^{n}+\nu^{n} \end{array},$$ (7)

where both ***η*** ^*n*^ and ***ν*** ^*n*^ are zero-mean stochastic variables, ***η*** ^*n*^ is the process noise, and ***ν*** ^*n*^ is the measurement noise. This formulation allows users to exploit process noise covariance as a tuning parameter. This, in turn, influences the convergence properties and tracking performance of the filter. For a system with stationary parameters, ***η*** ^*n*^ should be near zero as our confidence in the parameter estimates does not deteriorate over time. In our case, ${\hat{J}}^{n}$ changes with time and thus requires a positive definite covariance for the process noise. In general, larger values for the process noise covariance will not only lead to a quicker response to changes in ***J*** ^*n*^ but also to more noise in the estimate ${\hat{J}}^{n}$ . Conversely, small values for the process noise will produce less noise in the estimate of ${\hat{J}}^{n}$ but might introduce significant lag in the estimates.

We finally note the following detrimental but fundamental property that mimics the problem observed at the end of Section 4: the Jacobian estimation problem is not globally observable since the non-linear mapping defined in (6) is, in our case, a linear mapping with respect to ${\hat{J}}^{n}$ . The linear map is not bijective, preventing both global and local observability. However, the mapping retains the property of being *practically identifiable* (Wieland et al., 2021), a property that implies that the Jacobian ${\hat{J}}^{n}$ can be made observable by introducing sufficiently rich input–output data pairs. In our case, the parameters can be made observable by providing a sufficiently rich control input ${\dot{\phi }}^{n}$ . At the same time, due to the non-linear mapping being practically identifiable, it is difficult to infer any general guarantees on the stability, convergence, or correctness of the estimated Jacobians. This may have important implications for path planning and lower-level control, which should be carried out in such a manner as to render the system practically identifiable.

In summary, for both the algorithms proposed in this paper, the lack of persistently exciting inputs causes numerical and theoretical problems. As this paper is focused on proposing and characterizing these algorithms, how to mitigate this inherent problem is considered a future work.

## 6 Methods

**FIGURE 4:**
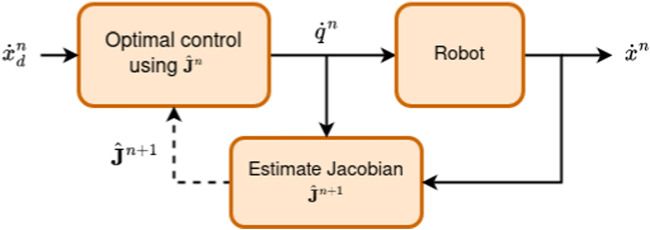
Digital render of a 5-link boa snake robot, with a soda can for scale reference.

This section outlines a series of simulation experiments designed to evaluate the performance and execution times of the two algorithms described in Sections 4 and 5 and their robustness to measurement noise. All experiments were performed using a snake robot simulator which was purpose-built for OAL research. The simulator is built upon the physics engine Coumans (2020). All experiments were performed on a simulated snake robot with 11 links that emulates a snake robot platform currently under development by the authors. A rendering of the robot is shown in Figure 4. Essential physical parameters of the simulated robot are given in Table 1. Three experiments were carried out to investigate different properties of the two Jacobian estimation algorithms. All three experiments share the same basic setup: the robot is set in a starting position *θ*_*N*_ = 0, ***r*** = **0**, and ***ϕ*** = **0** on an infinitely large plane populated by cylindrical obstacles, where **0** is the null vector of appropriate dimensionality. The obstacles have a radius of 50 mm, are fixed within the world frame, and are placed at regular intervals in two rows with coordinates given in meters by

$$\begin{array}{cc} {\left[\begin{array}{cc} 0.5k+0.25 & 0.1 \end{array}\right]}^{T} & \forall k\in \left\{-3,10\right\}\\ {\left[\begin{array}{cc} 0.5k & -0.1 \end{array}\right]}^{T} & \forall k\in \left\{-3,10\right\}\;. \end{array}$$ (8)

**FIGURE 5:**
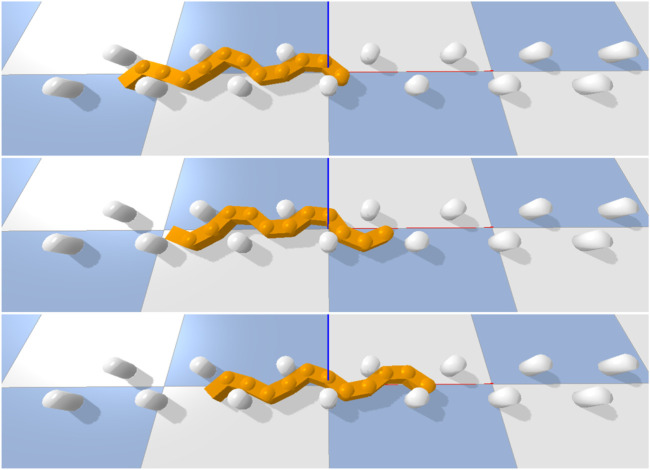
Simulated snake robot moving through its environment using a basic undulation pattern at three different time steps. Each of the colored squares on the underlying plane measures 1*m* ⋅1*m*.

The snake robot is actuated to perform an undulation pattern that creates propulsion by a rudimentary interaction with the obstacles without the need for feedback control. Note that this form of propulsion is only possible because the exact location and properties of the obstacles are known. We note that the position or geometry of the obstacle are *not* known to the two Jacobian estimation strategies and that it is only given for the sake of reproducibility of the experiments. The goal of the experiments is to examine the behavior of the Jacobian estimation, not the behavior of the control strategy. The design of a more complex control strategy leveraging the estimated Jacobians is considered a future work. The desired joint angles $\phi_{k}^{n}={\left[\begin{array}{cccc} \phi_{d,1}^{n}, & \phi_{d,2}^{n}, & \ldots , & \phi_{d,N_{j}}^{n} \end{array}\right]}^{T}$ are computed as

$$\phi_{d,k}^{n}=\frac{\pi }{3}\sin\left(4n\Delta_{t}-\frac{\pi }{3}k\right)\;,$$ (9)

where Δ_*t*_ is the time step of the simulation. The other parameters for the undulation pattern were chosen to create propulsion in the snake robot, for the given set of obstacles. A visualization of the snake moving using the pattern described in (9) through the obstacles described in (8) is shown in Figure 5A. For all three experiments, the simulation is run for a duration of 10s and a time step of $\Delta_{t}=\frac{1}{240}s$ . The three experiments are described as follows.

• *Experiment 1:* The Jacobian is estimated using measurements of ${\dot{r}}^{n}$ and ${\dot{\phi }}^{n}$ from the described simulation without further alteration.
• *Experiment 2:* Similar to experiment 1, except that the undulation of the robot is commanded to halt such that ${\dot{\phi }}^{n}$ rapidly approaches 0 at*t* = 5s and remains stationary until *t* = 7s, where the undulation is resumed.
• *Experiment 3:* Similar to Experiment 1, except that a measurement noise is applied to the measurement of ${\dot{r}}^{n}$ such that ${\dot{\tilde{r}}}^{n}={\dot{r}}^{n}+\delta_{r}$ , where $\delta_{r}∼N\left(0,\Sigma_{r}\right)$ and **Σ**_*r*_ = 0.1 ⋅ ***I*** is the covariance of *δ*_*r*_, with ***I*** being the identity matrix.

Ideally, the metric to measure the performance of the estimators would be to compare the estimated Jacobian $\hat{J}$ to the true Jacobian ***J***. As previously discussed, the true Jacobian is challenging to obtain, so other metrics of performance are used. We apply a metric commonly used in machine learning and see if our model produces the expected output from a known input. For each time step, a prediction is produced from our estimators based on the next input ${\dot{\phi }}^{n+1}$ and the current Jacobian ${\hat{J}}^{n}$ . The input ${\dot{\phi }}^{n+1}$ is applied to the snake robot and produces an output ${\dot{r}}^{n+1}$ . The true value of ${\dot{r}}^{n+1}$ is compared to that of the prediction from the estimators to evaluate their performance. The mean square error (MSE) is used to evaluate the error of the estimators.

**TABLE 1:** Summary of the most relevant physical parameters of the simulated robot.

Link width	84	mm
Axis–axis distance	130	mm
Link mass	500	g
Link friction coefficient	0.1	-
Joint torque	3	Nm

Both algorithms were implemented in Python, using *cvxpy* (Agrawal et al., 2018; Diamond and Boyd, 2016) for convex optimization and *filterpy* (Labbe, 2017) for the unscented Kalman filter and associated resources.

## 7 Results

All plots showing the results of experiments 1–3 are placed in the Appendix due to their size and visual complexity, but should be interpreted as a part of this section.

### 7.1 Experiment 1

**FIGURE 6:**
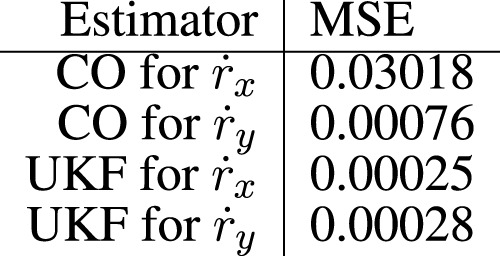
Comparison of the true $\dot{r}$ to the predicted values of $\dot{r}$ from the two estimators in experiment 1. Note that for the two topmost plots, the blue line showing the true ${\dot{r}}_{x}$ is mostly hidden behind the orange line showing the predicted ${\dot{r}}_{x}$ . The two bottom plots show the error between the $\dot{r}$ and the predicted values of $\dot{r}$ .

**FIGURE 7:**
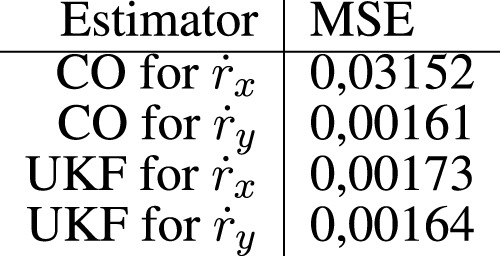
Mean square error (MSE) for the estimators in experiment 2

The data collected from experiment 1 can be visualized in Figures 6A–D. The CO-based algorithm provides decent predictions for the y-component of the head velocity vector ${\dot{r}}_{y}$ but diverges for the x-component of the head velocity vector ${\dot{r}}_{x}$ over time. The UKF-based algorithm predicts both components of $\dot{r}$ without diverging. The mean square error between the estimated $\dot{r}$ and the true value of $\dot{r}$ is shown in Figure 7.

### 7.2 Experiment 2

**FIGURE 8:**
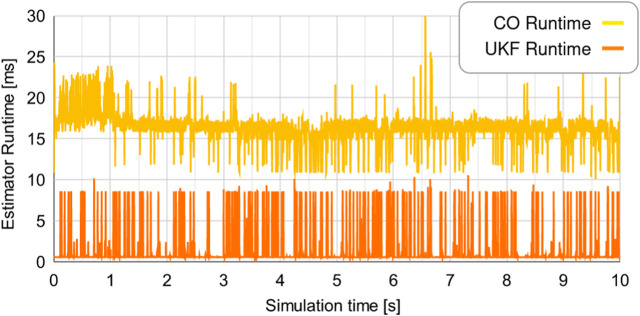
Comparison of the true $\dot{r}$ to the predicted values of $\dot{r}$ from the two estimators in experiment 2. Note that for the two topmost plots, the blue line showing the true ${\dot{r}}_{x}$ is mostly hidden behind the orange line showing the predicted ${\dot{r}}_{x}$ . The two bottom plots show the error between the $\dot{r}$ and the predicted values of $\dot{r}$ .

The data from experiment 2 are visualized in Figures 8A–D. As the robot becomes stationary shortly after *t* = 5s, the CO-based algorithm rapidly diverges. This is compliant with what was theorized in Section 4. As the robot continues its movement at *t* = 7s, the CO-based algorithm diverges further. As $\dot{\phi }\to 0$ , the UKF-based algorithm also displays stability issues. However, as the robot resumes moving at *t* > 7s, the UKF-based algorithm shows a higher degree of error until *t* = 7.3s but rapidly converges to the true value of $\dot{r}$ as *t* > 7.3s.

### 7.3 Experiment 3

**FIGURE 9:**
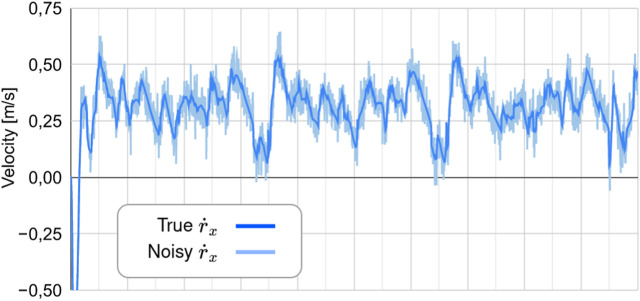
Comparison of the true $\dot{r}$ to the predicted values of $\dot{r}$ from the two estimators in experiment 3. Note that for the two topmost plots, the blue line showing the true ${\dot{r}}_{x}$ is mostly hidden behind the orange line showing the predicted ${\dot{r}}_{x}$ . The two bottom plots show the error between the $\dot{r}$ and the predicted values of $\dot{r}$ .

**FIGURE 10:**
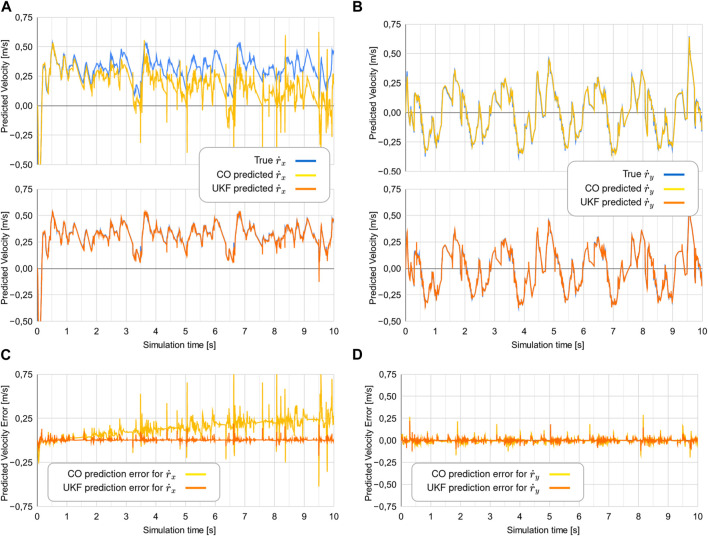
True ${\dot{r}}_{x}$ compared to the noisy signal used in experiment 3. **(A)** Comparison of the true r_x_ to the predicted values of r_x_, **(B)** Comparison of the true r_y_ to the predicted values of r_y_, **(C)** The error between the true r_x_ and the predicted values of r_x_, **(D)** The error between the true r_y_ and the predicted values of r_y_.

**FIGURE 11:**
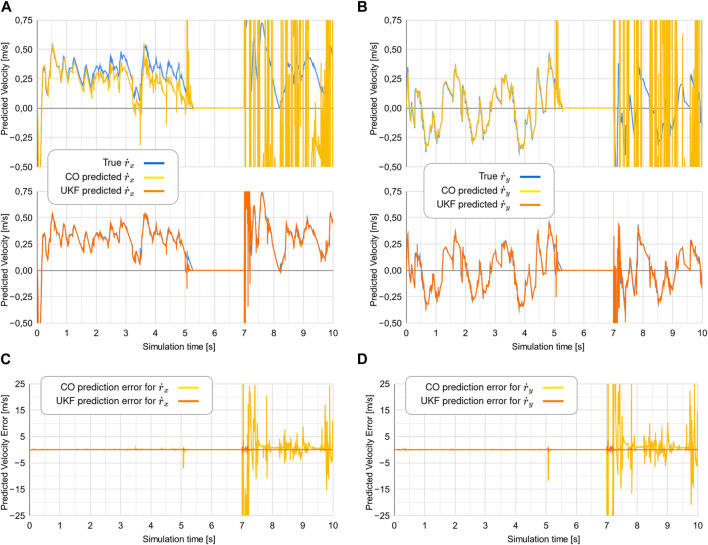
Mean square error (MSE) for the estimators in experiment 1. **(A)** Comparison of the true r_x_ to the predicted values of r_x_, **(B)** Comparison of the true r_y_ to the predicted values of r_y_, **(C)** The error between the true r_x_ and the predicted values of r_x_, **(D)** The error between the true r_y_ and the predicted values of r_y_.

The results from experiment 3 are shown in Figures 9A–D, with Figure 10 depicting a realization of the measurement noise about ${\dot{r}}_{x}$ . Similarly to what was seen in experiment 1, the CO-based algorithm diverges for ${\dot{r}}_{x}$ . To compare the two approaches from a quantitative perspective, we report the MSE of both algorithms in Figure 11.

### 7.4 Execution times

**FIGURE 12:**
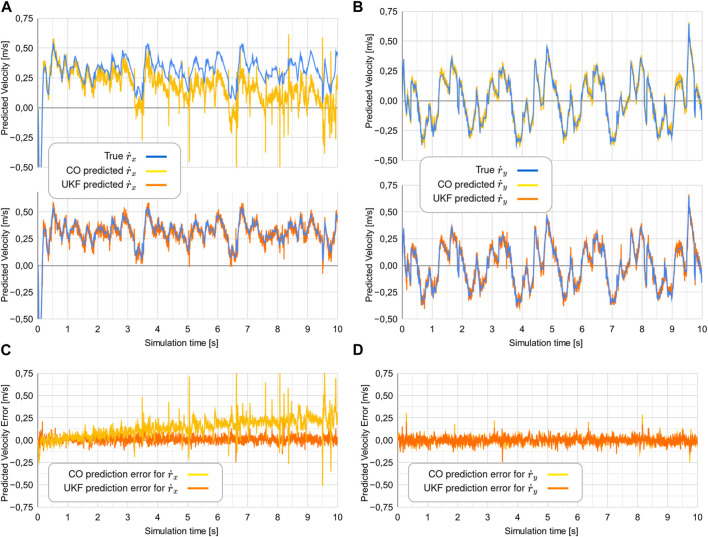
Computation time for each time step for the two estimators. **(A)** Comparison of the true r_x_ to the predicted values of r_x_, **(B)** Comparison of the true r_y_ to the predicted values of r_y_, **(C)** The error between the true r_x_ and the predicted values of r_x_, **(D)** The error between the true r_y_ and the predicted values of r_y_.

The execution times of both the algorithms are shown in Figure 12. The CO-based algorithm has an average computation time of 16.32 m for each time step, while the UKF-based algorithm has an average computation time of 1.25 m for each time step.

## 8 Discussion

### 8.1 Experiment 1

The UKF-based algorithm outperforms the CO-based algorithm by having a significantly lower MSE for both components of $\dot{r}$ . The error of both algorithms increases when there are large changes in $\dot{r}$ (e.g., at *t* = 8 where there is a near-instantaneous change in $\dot{r}$ ). This is expected, as a large unexpected change in velocity will rapidly change the true value of the Jacobian of the system.

### 8.2 Experiment 2

This experiment displayed the inherent stability issues of both algorithms, as theorized in Section 4. A key difference between them is seen in their behavior immediately after the robot resumes movement at *t* = 7*s*. The CO-based algorithm implements a strict equality bound. After the algorithm diverges when $\dot{\phi }\to 0$ and is restarted, the algorithm attempts to solve the constrained optimization problem to find ${\hat{J}}^{n+1}$ , based on an inaccurate estimate of ${\hat{J}}^{n}$ , leading to an inaccurate estimate of ${\hat{J}}^{n+1}$ . This problem is propagated into the next step of the algorithm. One simple solution to this issue would be to reset the values of the values of the Jacobian to ${\hat{J}}^{n}=0$ immediately after a halt. In contrast to the CO-based method, the UKF-based algorithm shows no degradation in performance after the halt compared to before the halt, except for a brief transient.

### 8.3 Experiment 3

The performance of the algorithms seems to degrade in a similar fashion when subjected to increasing measurement noise. The performance of the UKF-based filter can be tuned as described in Wan and Van Der Merwe (2000) by adjusting the values of the measurement and process noise covariance matrices. The ratio of the elements in these two matrices controls the trade-off between the filter’s ability to rapidly respond to sudden changes in state and the filter’s robustness to noise.

### 8.4 Analysis of execution times

The higher computation time for the CO-based algorithm is likely due to the estimator being based on a numerical solver; the *cvxpy* platform uses the open-source OSQP, SCS, and ECOS solvers Agrawal et al. (2018). The lower computation time of the UKF-based algorithm is likely because it is based on basic matrix computations and decompositions instead of numerical optimization. It may be possible to improve the average computation time for both algorithms through code optimization; such optimization has not been part of this study. Due to the fundamental differences in the two algorithms, however, we expect our qualitative comparison to survive such improvements.

### 8.5 Possibilities for generalization

While this paper focuses on Jacobian matrix estimation for planar snake robots, both algorithms are adaptable to a 3-dimensional scenario by increasing the dimensionality of the measurement vector ${\dot{r}}^{n}$ from $R^{2}$ to $R^{3}$ . Care should be taken during this process, as the number of unobservable variables will increase and the issues with convergence and observability will likely worsen as the dimensionality of the measurement vector increases. Both algorithms presented in this paper are easy to adapt to other kinds of problems as they require little or no information about the dynamics of the system they are applied to. The proposed UKF-based algorithm may be relevant to the field of soft robotics or constrained robotics with unknown kinematics. Generally, the UKF-based algorithm may be useful for any system that can be modeled as the general parameter estimation problem in (6).

## 9 Conclusion

Jacobians capture the complex interactions of a planar snake robot. It models a constrained kinematic system, with its surrounding obstacles, by representing a mapping between the robot’s joint velocities or torques on one hand and its movements or interaction forces on the other hand. A constrained snake robot’s Jacobian matrix may, thus, be used as part of an obstacle-aided locomotion (OAL) control scheme to allow the robot to utilize the surrounding obstacles and its many joint actuators to move efficiently through the environment. We have shown how Jacobians can be estimated from proprioceptive (internal) measurements and proposed and tested two different strategies for obtaining such estimates.

The two methods, one based on constrained optimization concepts (CO-based) and one based on unscented Kalman filtering techniques (UKF-based), have been shown to perform quite differently. The UKF-based algorithm has a significantly lower computation time than the CO-based algorithm, while at the same time giving more accurate predictions of the end-effector velocity for a variety of simulation scenarios. Importantly, the UKF-based algorithm performs much better in scenarios where the snake halts, in which case the CO-based algorithm is plagued by divergence issues. This paper presents and analyzes the algorithms from numerical perspectives, but we foresee further research into the stability and convergence properties of both algorithms as they are both practically identifiable. These properties are important as they would provide some guarantee to the correctness of the estimates, which, in turn, could prove important in the design of control strategies that leverage these estimates.

Further research should also be dedicated to devising and comparing other types of methods for the purpose of finding which one is the most suitable for OAL. Recent advances in the modeling of snake robots using geometric algebra (Hrdina et al., 2016) may prove useful in explicitly modeling the kinematics of a robot’s interaction with obstacles, without the need for an estimation-based approach.

While this paper focuses on Jacobian matrix estimation for planar snake robots, the proposed algorithms can readily be adapted to a range of problems within robotics where the kinematics of a system is impractical or impossible to obtain analytically.

## Data Availability

The raw data supporting the conclusion of this article will be made available by the authors, without undue reservation.
